# Transcatheter closure of PDA: how the pathway changed from classical fluoroscopy approach

**DOI:** 10.3389/fcvm.2026.1811280

**Published:** 2026-05-21

**Authors:** Sisca Natalia Siagian, Elsa Hedia Panjaitan, Christianto Christianto

**Affiliations:** 1 Division of Pediatric Cardiology and Congenital Heart Disease, Department of Cardiology and Vascular Medicine, Faculty of Medicine Universitas Indonesia/National Cardiovascular Centre Harapan Kita, Jakarta, Indonesia; 2 Faculty of Medicine Universitas Indonesia, Jakarta, Indonesia

**Keywords:** echocardiography, fluoroscopy, patent ductus arteriosus, radiation, transcatheter closure

## Abstract

Patent ductus arteriosus (PDA) is a common congenital cardiac defect traditionally managed by surgical and transcatheter closure under fluoroscopic guidance. Over recent years, significant advances in imaging modalities, device technology, and procedural planning have transformed the pathway for PDA closure, reducing reliance on classical fluoroscopy-based techniques. This review outlines the evolution of transcatheter PDA closure from conventional fluoroscopic approaches to contemporary strategies incorporating echocardiographic guidance, hybrid imaging, and radiation-sparing protocols. These changes have enhanced procedural safety, reduced radiation exposure to patients and operators, shortened procedure times, and expanded eligibility to younger and higher-risk populations. Understanding this evolving pathway is essential for optimizing outcomes and guiding future practice in transcatheter PDA closure.

## 1 Introduction

Patent ductus arteriosus (PDA) is a common congenital heart defect, representing 7%–10% of all congenital heart conditions, making it the third most prevalent. It may occur as an isolated defect, coexist with other simple acyanotic lesions, or be part of more complex critical cyanotic defects, such as duct-dependent lesions. PDA can be closed either through surgical or nonsurgical methods. Surgical closure often requires extensive medication, a longer hospital stay, and can lead to complications or scarring, which some patients may find troublesome. As a result, transcatheter interventions have become the preferred treatment. However, these interventions come with their own risks, such as radiation exposure for both patients and operators. Developing alternative methods that reduce complications and minimize radiation is highly desirable (1–5).

## 2 Embryology, pathophysiology, and classification

PDA is a condition in which the ductus arteriosus, a vessel connecting the descending aorta and the left pulmonary artery, fails to close after birth. The ductus arteriosus originates from the left sixth aortic arch, and plays a critical role in fetal circulation, shunting blood away from the developing lungs. The ductus normally undergoes functional closure within the first 24–48 h of life, followed by anatomical remodeling into the ligamentum arteriosum within 2–3 weeks. Failure of this process results in persistent ductal patency (1, 6).

Like other left-to-right shunt lesions, PDA can lead to significant hemodynamic disturbances, causing complications and potentially fatal outcomes if left untreated. The mortality rate for patients with hemodynamically significant PDA (hsPDA) reaches 0.49% in children aged 2–19 years, and 1.8% in those over 20 years old, with 30% of these deaths attributed to congestive heart failure. The higher systemic vascular resistance compared to pulmonary vascular resistance drives a continuous left-to-right shunt from the aorta to the pulmonary artery, the magnitude of which depends on ductal size and vascular resistances. Over time, significant shunting leads to pulmonary overcirculation and increased left heart volume load, resulting in left ventricular dilation and eventual heart failure. Chronic exposure to increased pulmonary blood flow may also promote pulmonary vascular remodeling, with rising pulmonary arterial pressures that can progress to pulmonary arterial hypertension and right heart failure. In advanced cases, reversal of shunt direction (Eisenmenger physiology) may occur. Additionally, even small PDAs can cause significant complications due to turbulent blood flow that irritates the surrounding tissues, increasing the risk of infective endarteritis and arrhythmias. Therefore, PDA should be actively monitored and managed to prevent long-term sequelae (1, 3, 6, 7).

**Figure 1:**
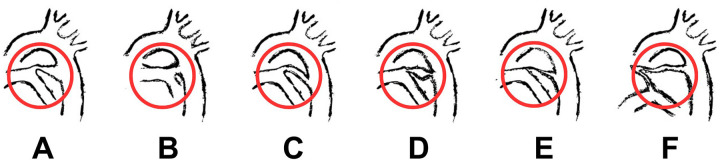
Description of the modified krichenko classification. Type **(A)** is a conical duct with a prominent aortic ampulla and constriction at the pulmonary artery end. Type **(B)** is a window duct, short and wide, with a mild constriction at the aortic end. Type **(C)** is a tubular duct without any constrictions. Type **(D)** is a saccular duct with constrictions at both the aortic and pulmonary artery ends. Type **(E)** is an elongated, narrow ductus with a constriction at the pulmonary artery end. Type **(F)** is a fetal type duct (1, 9).

The classification of PDA is important for determining the most appropriate intervention. Krichenko's classification categorizes PDA based on angiographic morphology into five types (A–E). Phillips et al. later modified Krichenko's classification by introducing Type F, a fetal-type duct found exclusively in premature infants. Type F is characterized by its wide, long shape with minimal tapering from the aortic to pulmonary end. Although it shares similarities with Type C, Type F is distinguished by tortuosity near the pulmonary artery end, giving it a “hockey-stick” appearance, unlike the tubular form of Type C. Figure 1 illustrates the difference between PDA types (8, 9).

## 3 Which PDA should be closed?

For reasons aforementioned, all cases of PDA should be carefully evaluated to determine suitability for closure, with consideration of hemodynamic significance and procedural risk vs. benefit. Even small PDAs should be assessed for suitability of closure to prevent potential infections. For moderate to large PDAs, closure may be critical because these can lead to significant hemodynamic changes that may eventually result in heart failure. Echocardiographic assessments help determine the need for closure, with key criteria relating to the ductal size (diameter) or shunt pattern, evidence of volume overload, pulmonary overflow, or systemic/tissue hypoperfusion. In premature neonates, a PDA diameter of 1.5 mm or a PDA/left pulmonary artery ratio of ≥0.5, along with a growing or pulsatile shunt pattern, indicates a hemodynamically significant PDA (hsPDA) (8–10). Additional signs include systemic overcirculation, indicated by a left atrium/aortic ratio >1.4 or a left ventricular output greater than 300 mL/kg/min; increased pulmonary arterial flow, evidenced by a left pulmonary artery end-diastolic velocity >20 cm/s; retrograde blood flow in the descending aorta; low antegrade flow during systole or diastole; and absent or reversed end-diastolic flow in organs such as the middle cerebral, superior mesenteric, or renal arteries, which signify tissue hypoperfusion and steal syndrome (11, 12).

According to the European Society of Cardiology (ESC) Guidelines for the management of adult congenital heart disease, PDA closure is recommended for all cases with signs of left ventricular (LV) volume overload, pulmonary arterial hypertension (PAH) with a pulmonary artery pressure (PAP) <2/3 of systemic pressure, or pulmonary vascular resistance (PVR) <2/3 of systemic vascular resistance (SVR). Device closure is the preferred method of intervention when technically feasible or in cases of PAH with PAP and PVR >2/3 of SVR, but still with a net left-to-right (L–R) shunt (fractional ratio >1.5), and positive reactivity tests (13). Additionally, small PDAs with a continuous murmur, normal LV, and normal PAP, are also candidates for closure. However, in silent PDAs, Eisenmenger syndrome, and cases where exercise induces lower-limb desaturation, the defect should not be closed (12, 14).

## 4 History of PDA closure

The approach to managing PDA has evolved significantly over the years and continues to be a topic of discussion in pediatric cardiology. Initially, PDA closure, especially in preterm infants, was primarily achieved through pharmacological methods using non-steroidal anti-inflammatory drugs (NSAIDs) such as indomethacin, ibuprofen, and acetaminophen. These medications are still commonly used today for preterm infants, successfully closing PDA in 60%–80% of cases. Pharmacological therapy remains a subject of ongoing research, with ongoing investigations into the best indications, timing, drug dosages, choices, and potential side effects (8, 10, 11, 15, 16).

Surgical ligation of the ductus arteriosus marked a significant milestone in the treatment of PDA, especially when pharmacological approaches are ineffective or not recommended. Surgical closure involves ligating or cutting the PDA through a left posterior lateral thoracotomy or, more recently, using video-assisted thoracoscopic surgery (VATS). Although early surgical closure was associated with considerable morbidity and mortality, these techniques have improved considerably over time. Studies now report a complete closure rate exceeding 94%, with a relatively low mortality rate of 0%–2%. Despite being effective and safe, surgical closure is linked to higher morbidity, postoperative pain, longer hospital stays, visible scarring, and potential complications. The most common complications include pneumothorax, bleeding, and recurrent laryngeal nerve injury (10, 11).

The limitations of surgery led to the development of transcatheter techniques for PDA closure, which have become the preferred method of treatment for many patients who meet the criteria. The occlusion devices used for transcatheter PDA closure have evolved substantially over the decades. Early attempts in the 1960s–70s used plug devices made from Ivalon sponges and umbrella-like systems such as the Rashkind occluder. Although these devices demonstrated the feasibility of nonsurgical closure, their use was limited by complex deployment and large delivery systems. In the 1990s, the self-adjustable PDA device, later modified into the buttoned device, allowed delivery through smaller sheaths but was associated with relatively high rates of residual shunting, device instability, endarteritis, and other complications. Subsequent development of coil embolization devices addressed several of these limitations by allowing delivery through small catheters and enabling repositioning or retrieval before release while minimizing obstruction to adjacent vessels. However, coils carried a risk of embolization and their use was limited to favorable ductal anatomy, most commonly Krichenko type A ducts. The introduction of the Amplatzer Duct Occluder (ADO) represented a major advancement, providing more reliable occlusion across a wider range of duct sizes and morphologies. Continued refinements have since produced devices with smaller delivery profiles, improved conformability to various duct types, and greater stability, enabling safe closure even in very small infants, including those weighing less than 1 kg (10, 11, 17, 18).

## 5 The classic transcatheter closure

Transcatheter closure of PDA involves the insertion of a catheter or delivery sheath to cross the ductus arteriosus, either anterogradely via the femoral vein or retrogradely through the femoral artery. After crossing the ductus, an appropriate closure device is placed inside the duct to occlude it. Traditionally, this procedure is guided by fluoroscopy and contrast use, which help define the defect's profile and allow for accurate device placement during and after the procedure. Prior to device insertion, biplane angiography in lateral and/or right anterior oblique (RAO) views is performed. Based on these angiographic measurements, the appropriate device and size are selected. Fluoroscopy is then used to guide the catheter and position the device within the PDA. A follow-up angiogram is done later to assess device positioning and check for any residual shunting (10–12, 18, 19).

Fluoroscopy-guided techniques have been the standard for percutaneous PDA closure for many years. However, concerns persist about the risks associated with radiation and contrast exposure for both patients and healthcare providers (5, 20). This is particularly concerning for pediatric patients, who are more susceptible to the stochastic risks of radiation, such as cancer, due to their higher tissue radiosensitivity and longer life expectancy following the procedure (21, 22). Some studies show substantial variation in radiation doses during PDA closure procedures (23–25), but the stochastic effects of radiation are difficult to predict (25–27). These effects are independent of a dose threshold, though their frequency increases with higher radiation exposure (28–30). To minimize such risks, the principle of As Low As Reasonably Achievable (ALARA) remains strongly advocated by institutions such as the International Commission on Radiological Protection. Additionally, the long-term radiation exposure risks to operators are significant, with studies indicating increased risks of skin injuries, cataracts, hair loss, and even malignancies among healthcare workers performing these procedures routinely (31). Furthermore, the use of heavy lead aprons as personal protective equipment (PPE) has been linked to chronic issues for operators, including back problems (32).

## 6 Zero fluoroscopy

These limitations have encouraged cardiologists to look for better ways to perform non-surgical defect closure. In 2000, Peter Ewert et al. first introduced a zero-fluoroscopy approach for percutaneous atrial septal defect (ASD) and patent foramen ovale (PFO) closure (33). They showed that the procedure was feasible using echocardiographic guidance alone, reporting successful closure in 19 of 22 patients. This success was supported by operator experience and the availability of devices that were easy to handle. However, after this initial report, the technique was not widely adopted. Interest in the method increased in 2007, when Xiangbin Pan and his team reported successful zero-fluoroscopy procedures in a larger number of patients and published their experience in greater detail (34). Pan et al. have also provided a comprehensive guideline for this approach (35).

Nowadays, the zero-fluoroscopy technique has been increasingly adopted, although the classical fluoroscopy-guided approach is still more commonly practiced. The recommendation for zero-fluoroscopy remains debated, and its adoption often depends on each institutional experience. Despite this, zero-fluoroscopy interventions represent a promising development in the field, with several recent studies reporting favorable outcomes and demonstrating safety and effectiveness comparable to conventional techniques (36–41). A meta-analysis published in 2025 also reported similar success rates and clinical outcomes between zero-fluoroscopy and classical approaches (42). As the development and wider adoption of this approach are relatively recent, further studies—particularly randomized controlled trials—are needed to better define its role in routine clinical practice.

Our center began adopting the zero-fluoroscopy method in 2018, initially for ASD closure, followed by PDA and ventricular septal defect (VSD) closure. We now routinely perform this procedure alongside the classical technique and have reported our results in several publications. In our experience, the zero-fluoroscopy technique addresses concerns related to radiation exposure and contrast use while providing comparable safety and effectiveness, consistent with findings from other studies (42–50). However, we have also identified several limitations that may reflect concerns raised by other centers regarding the adoption of this method. First, we observe longer anesthesia and procedural times when interventions are guided solely by echocardiography. Furthermore, in a zero-fluoroscopy setting, the interventional pediatric cardiologist must perform the procedure based on information conveyed by the echocardiography operator (also called the marshaller), effectively relying on the “eyes” of the marshaller. This requires a steeper learning curve and very close teamwork between the interventionist and the marshaller, and may lead to miscommunication or misunderstanding, particularly when experience is limited or when the team has not yet developed good coordination.

We would also like to discuss the differences between the antegrade and retrograde approaches for percutaneous PDA closure. The antegrade approach requires the catheter to be guided through a longer and more tortuous pathway, making the procedure technically more demanding. In contrast, the retrograde approach follows a more direct route to the PDA and is often easier to perform. Nevertheless, it requires arterial access and carries a higher risk of vascular complications, including acute limb ischemia. For this reason, the retrograde approach is generally limited to cases using smaller devices. In our practice, especially in pediatric patients, we prefer the antegrade approach to minimize the risk of vascular complications while maintaining procedural safety. Although technically more challenging, this approach allows more stable device delivery and aligns well with strategies aimed at reducing fluoroscopy use.

## 7 The “not really” classic transcatheter closure (minimal fluoroscopy without angiography)

We therefore developed an approach that seeks a balance between the classic and zero-fluoroscopy techniques. In this method, an antegrade approach with minimal fluoroscopy and without contrast administration (no angiography) is applied. The procedure begins with assessment of the PDA and its dimensions using transthoracic and transesophageal echocardiography (TTE and TEE) to determine suitability for device closure and to select the appropriate device. Previous studies have proved both TTE and TEE have good accuracy in determining defect size. Next, fluoroscopy is used to guide catheter advancement from the inferior vena cava to the right atrium, right ventricle, pulmonary artery, across the PDA, and into the descending aorta. We found this step to be the most challenging aspect of a fully zero-fluoroscopy approach, as difficulty in tracking the catheter through this tortuous pathway may compromise procedural safety and prolong procedure time (50). Fluoroscopy is also used to perform diagnostic RHC in this stage if indicated. No angiography is performed. Once the device is positioned, deployment and post-release evaluation are guided entirely by echocardiography.

**Figure 2:**
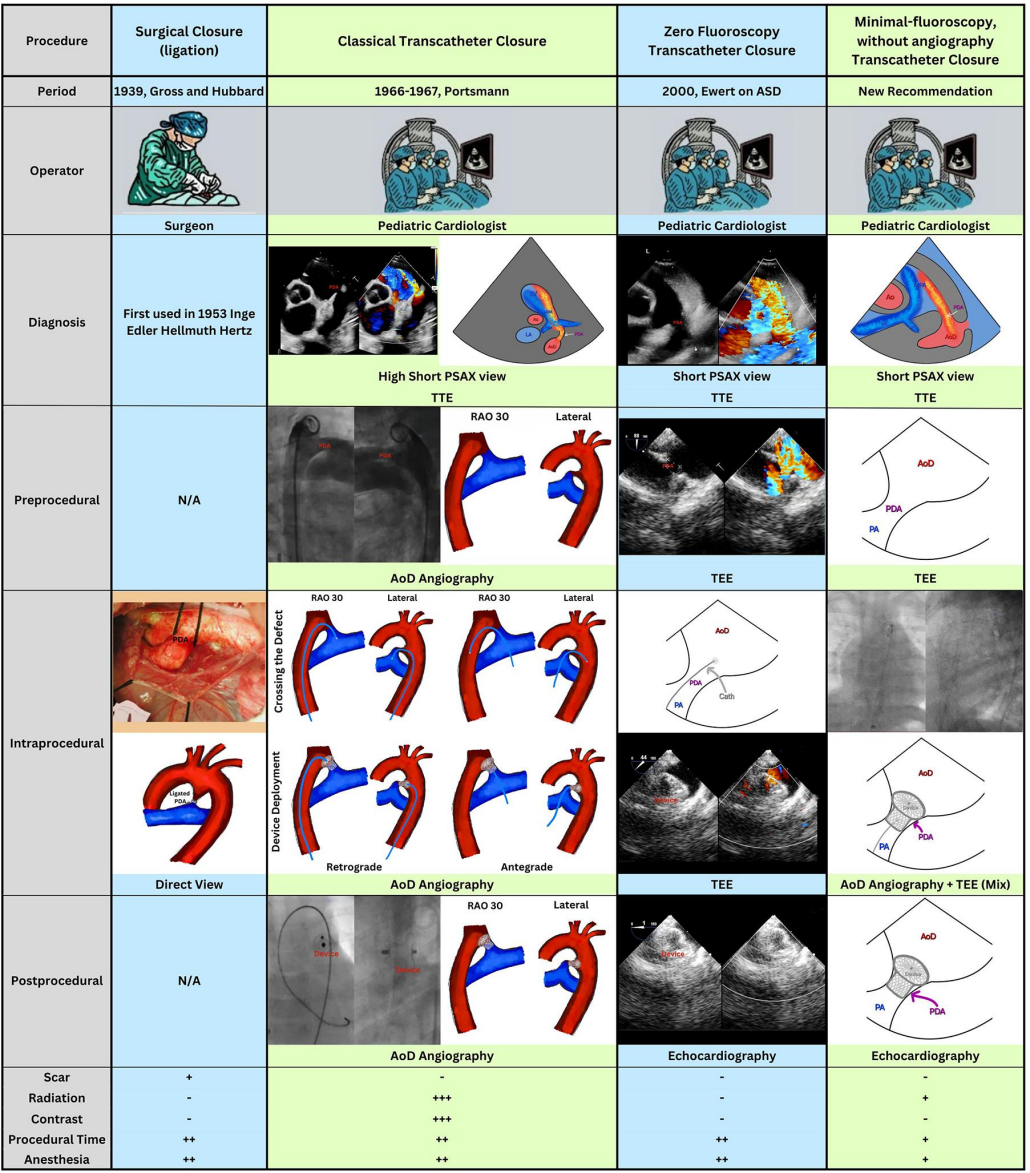
A summary comparing the evolution of PDA closure management, from surgical ligation to “minimal-fluoroscopy without angiography” approaches performed without contrast.

Currently, most transcatheter PDA closure procedures in our center are performed using this “minimal-fluoroscopy-without-angiography” technique. To date, we have applied this approach in 80 patients, including those with large PDAs and PDA associated with pulmonary arterial hypertension, achieving a 100% successful closure rate with no observed cases of device dislodgment or significant complications.We suggest that this strategy may offer a practical balance between minimizing radiation and contrast exposure—similar to the goals of zero-fluoroscopy techniques—while maintaining procedural safety and enabling efficient and accurate device placement. However, we would like to highlight that this technique still involves a learning curve and requires experienced interventional pediatric cardiologists and close coordination with the echocardiography operator (marshaller). Figure 2 summarizes the comparison between PDA closure methods.

We are planning a comparative study of different percutaneous PDA closure techniques, which we hope to report these data in detail in future publications. Ultimately, this paper aims to reflect the physician's ongoing pursuit of improved approaches to patient care, recognizing that patients remain the central focus and the point of impact of every intervention we undertake.

## 8 Conclusion

Advances in device technology, imaging modalities, and the expertise of interventional pediatric cardiologists and echocardiographers have facilitated the adoption of a minimal-fluoroscopy, without angiography approach to transcatheter PDA closure. These developments have improved procedural safety, significantly reduced radiation exposure for both patients and operators, shortened procedure duration, and broadened applicability to younger and higher-risk patients. A clear understanding of this evolving strategy is crucial for optimizing clinical outcomes and informing future practice in transcatheter PDA closure.

## Data Availability

The original contributions presented in the study are included in the article/Supplementary Material, further inquiries can be directed to the corresponding author.
